# Network meta-analysis of acupuncture for tinnitus

**DOI:** 10.1097/MD.0000000000035019

**Published:** 2023-09-29

**Authors:** Lin Ji, Haopeng Zhang, Lihua Wang, Ziming Yin, Jingtu Cen, Yu Guo

**Affiliations:** a Department of Otolaryngology, Shanghai Municipal Hospital of Traditional Chinese Medicine, Shanghai University of Traditional Chinese Medicine, Shanghai, China; b School of Health Science and Engineering, University of Shanghai for Science and Technology, Shanghai, China.

**Keywords:** acupuncture, network meta-analysis, tinnitus

## Abstract

**Objective:**

To provide evidence for medical management of tinnitus based on an assessment of the evidence concerning the effectiveness of acupuncture as a treatment for tinnitus using network meta-analysis (NMA).

**Methods:**

We conducted a systematic literature review by searching 8 national and international databases (inception to February 2023) for randomized controlled trials (RCTs) for tinnitus. Only RCTs that recruited participants aged over 18 and diagnosed with tinnitus, and that evaluated acupuncture or acupuncture in combination with conventional western medical therapy were included. We used response rate and tinnitus handicap inventory (THI) to examine efficacy. We conducted NMA with random effects, and the rate ratio or mean difference with its 95% credible interval was calculated. In addition, we ranked all treatments via their SUCRA and assessed the quality of evidence according to the GRADE criteria.

**Results:**

A total of 2575 patients were included in the study. The main findings of the current NMA were that acupoint injection combined with warm acupuncture was the most effective for response rate, followed by warm acupuncture and acupoint injection combined with western medical treatment. Acupuncture combined with western medical treatment was the most effective for THI, followed by electroacupuncture combined with warm acupuncture and acupuncture combined with moxibustion.

**Conclusion:**

Acupuncture seems to be a better trend treatment for tinnitus. Further rigorous RCT studies that include direct comparisons for different acupuncture-related treatments are encouraged to provide the most promising evidence for patients with tinnitus.

**Protocol registration:**

CRD42023398745.

## 1. Introduction

Tinnitus refers to the sensation of one or more sounds produced without an external sound source. In epidemiological studies worldwide, approximately 10% to 15% of adults will suffer from tinnitus, of which approximately 20% require clinical intervention.^[1]^ In China, due to the lack of large-scale epidemiological surveys, tinnitus prevalence is estimated to range from 7.8% to 30.4%, and shows a gradually increasing trend.^[2]^ Tinnitus is often accompanied by hearing loss, or worse, sleep disorder, depression, anxiety, irritability, restlessness, and other emotional abnormalities, ultimately affecting their quality of life. Not surprisingly, tinnitus imparts an enormous economic and social burden. It is thought that tinnitus is caused by changes in the neuronal network including auditory and non-auditory systems.^[3]^ Activation of non-auditory brain areas, such as the limbic system, may lead to misperception of tinnitus and ensuing negative emotions which can intensify the sensation of tinnitus and cause more negative emotions, ultimately leading to a “vicious circle,” which in turn reinforce the perception of tinnitus.^[4]^ Although many treatment approaches such as counseling, cognitive behavioral therapy, tinnitus retraining therapy, hearing aids and pharmacological therapy are available, the efficacy of most interventions for tinnitus remains unclear.

Acupuncture as an important part of complementary and alternative medicine has a long history in the treatment of tinnitus, which has been described and understood in detail as far back as the Huangdi Neijing, 2 thousand years ago. Clinical and experimental studies have also confirmed that acupuncture plays an important role in the neurophysiological modulation of the olivocochlear nucleus, the non-classical ascending auditory pathway, neural plasticity and the somatosensory system.^[5]^ An increasing number of studies have been published to support that acupuncture can effectively relieve the symptoms caused by tinnitus in patients, reduce the loudness and disability of tinnitus, and improve quality of life,^[6–8]^ but some studies have contradictory results.^[9,10]^ There are many systematic reviews have investigated acupuncture for the treatment of tinnitus,^[11–13]^ however, those studies only compare their own therapeutic results against their own targeted treatments, and the current guidelines declined the recommendation of acupuncture interventions for tinnitus management.^[14,15]^ Comparisons of the efficacy of multiple acupuncture interventions are therefore necessary, but it is difficult to conduct large-scale randomized controlled trial. NMA is conducted based on statistical method to calculate the direct and indirect comparison between multiple treatments. Considering these issues, in this paper, we conducted an NMA of the published randomized controlled trials (RCTs) to compare the relative efficacy of different acupuncture treatments with conventional western medicine for tinnitus patients to provide better guidelines for physicians to select the most effective treatment in clinical practice.

## 2. Methods

### 2.1. General guidelines of the study

We followed the latest preferred reporting items for systematic reviews and meta-analysis (PRISMA) 2020 guideline (Suppl. Table 1, http://links.lww.com/MD/J674) and AMSTAR2 (Assessing the method logical quality of systematic reviews) Guidelines to accomplish the current NMA.^[16,17]^ The current study had been registered in PROSPERO (CRD42023398745).

### 2.2. In search of strategies

We conducted a systematic literature review by searching PubMed, Embase, Cochrane Library, Web of Science, China National Knowledge Infrastructure, China Biomedical Literature Database, China VIP Database, and the Wanfang Database (inception to February 2023) for RCTs for tinnitus. No language restriction was used. Manual searches were also used for collecting papers. The search used subject headings combined with free words. The search terms included the “acupuncture therapy,” “acupuncture treatment,” “electroacupuncture,” “moxibustion acupuncture,” “Tinnitus,” “Ringing-Buzzing-Tinnitus,” “Ringing Buzzing Tinnitus,” “Tinnitus, Clicking,” “Clicking Tinnitus,” “Tinnitus, Leudet,” “Leudet Tinnitus,” “Tinnitus, Leudet’s,” “Leudet’s Tinnitus,” “Tinnitus, Leudets,” “Tinnitus, Subjective” and “Subjective Tinnitus.”

### 2.3. Data inclusion standards

Studies were included based on the following criteria: the study must be a randomized controlled clinical trial, and patients with idiopathic tinnitus or primary tinnitus were clearly diagnosed with reference to the consensus of 2012 tinnitus experts and diagnostic criteria of idiopathic tinnitus in interpretation and the definition of addressed in the important guideline by Tunkel, D.E. (2014)^[5,18]^; the interventions administered in the treatment group of the original study were consisted of various acupuncture-related therapies including acupuncture, moxibustion, electroacupuncture, acupoint injection, warm acupuncture, a combination of any 2 of these methods, or a combination of any of these methods with any extra conventional western medical treatment; additionally, the interventions for the control group were conventional western medical treatment or acupuncture therapy that differed from that of the treatment group; the study must have a statistical result; and the study must have a sample size of more than 20 cases.

### 2.4. Data exclusion standards

The exclusion criteria were: not a clinical trial; not a control study; studies that have identified tinnitus with a specific cause; studies in which no acupuncture is the dominant treatment method for patients; the data report is incomplete and the data cannot be used; repeat the research content; and studies that have low readability or trustworthiness.

### 2.5. Literature screening and data extraction

Two reviewers independently searched the databases and evaluated eligible articles for inclusion. Disagreement was resolved by discussion with a third reviewer. The following information was extracted independently by the reviewers: author name, publication year, number of research cases, patients’ ages, disease duration, treatment and control intervention (for acupuncture: type, needling location, needle retention time, and number of sessions of treatment; for western medical treatment: type), outcome measures and follow-up.

### 2.6. Outcomes

#### 2.6.1. Primary outcome.

The primary outcome was change in the response rate related to the treatment in patients with tinnitus. The response rate was defined on the basis of the criteria applied in the included studies. The THI count was secondary outcome.

### 2.7. Study appraisal

The Cochrane risk of bias assessment tool was used by 2 researchers (Lin Ji and Haopeng Zhang) to assess for the methodological quality of each study.^[19]^ The domains were assessed and determined to be at low, unclear or high risk of bias. Each study was given an overall risk of bias grade.

### 2.8. Statistical analysis

The NMA was performed using Stata14’ s mvmeta, network packages. We estimated the mean difference (MD) with a 95% confidence interval (95%CIs) for continuous variables. For the categorical variables, we used the rate ratio (RR) and 95%CIs.

A diagram of the evidence network was generated. The size of the dots in the figure indicates the sample size of the intervention, and the thickness of the line indicates the amount of direct evidence between the interventions. If there was a closed loop, an inconsistency model was used to evaluate the degree of consistency between the results of the direct comparison and the indirect comparison. If there was no closed loop, the consistency model was used for the analysis. If the *P* > .05, the inconsistency was low. If the *P* < .05, the inconsistencies were reported, and the evaluation results were treated with caution.

We calculated the relative ranking probabilities of the treatment effects of all treatments for the target outcomes to provide additional information for clinical applications. In short, the surface under the SUCRA represents the percentage of the mean rank of each treatment relative to the best imagined intervention with no certainty. When the area under the curve was larger, the treatment deserved a higher rank of benefit in the treatment of tinnitus.

## 3. Result

### 3.1. Literature search and basic features

**Figure 1.**
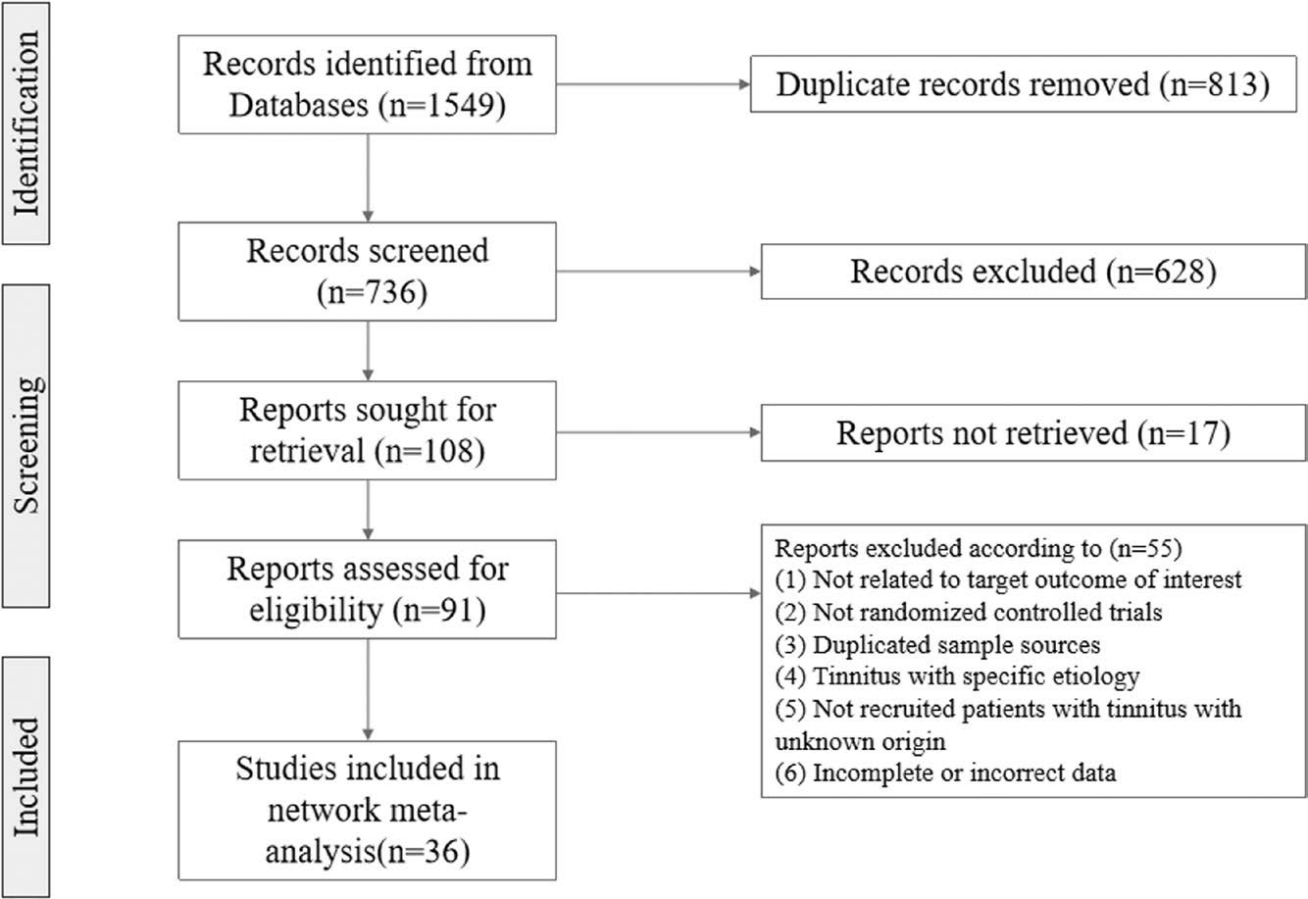
Flowchart of searching and screening for the studies.

The preliminary search yielded 1549 articles, and a total of 108 articles were considered for full-text review after the screening. Finally, 36 articles were included in the current study (Fig. 1). A total of 2575 patients (1316 in the intervention group and 1259 in the control group) were included in the study, all of whom met the diagnostic criteria of idiopathic tinnitus or primary tinnitus. The baseline characteristics of the included articles are summarized in Suppl. Table 2, http://links.lww.com/MD/J675.

### 3.2. Quality evaluation of the included articles

**Figure 2.**
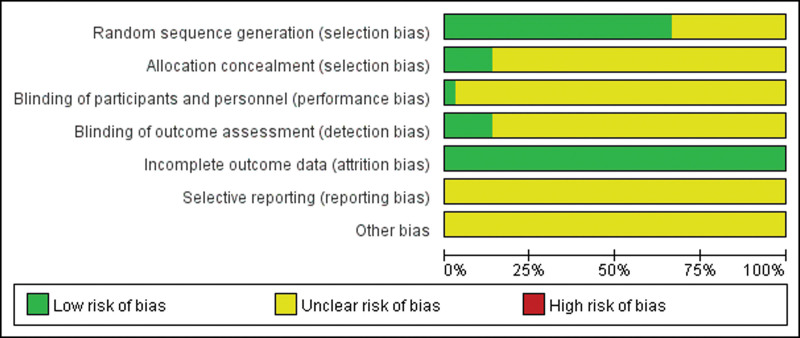
Risk of bias graph.

**Figure 3.**
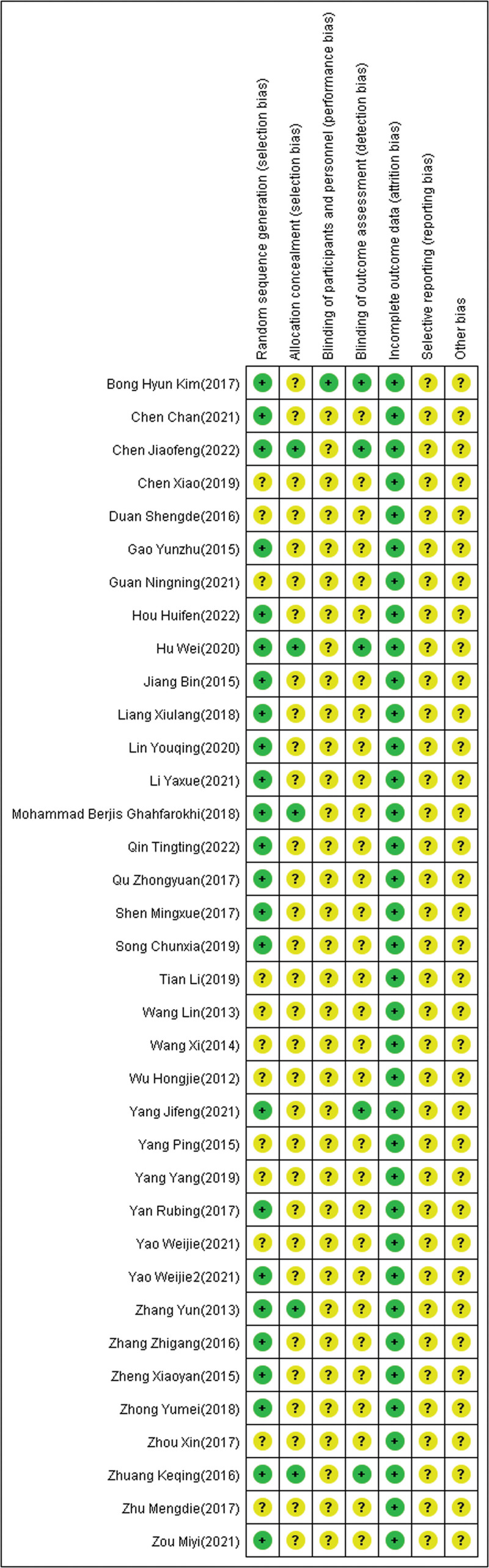
Potential risk of bias of each included study.

**Figure 4.**
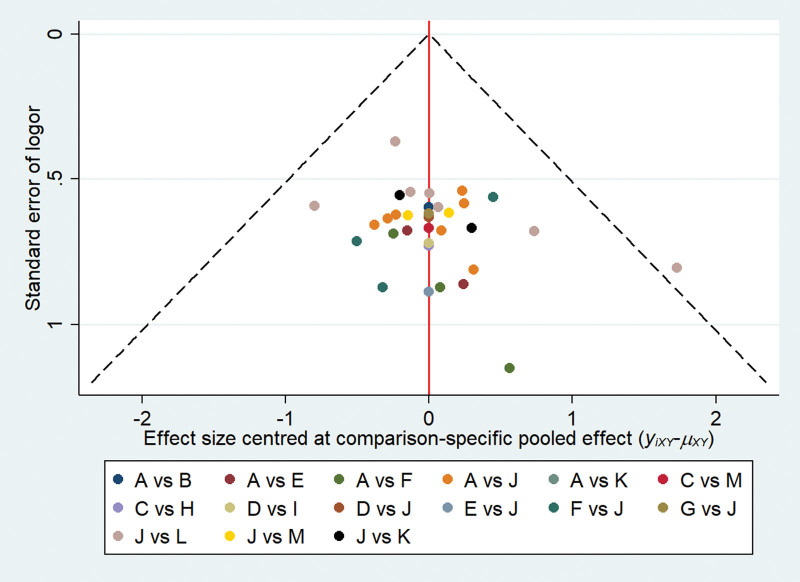
Funnel plot of response rate.

**Figure 5.**
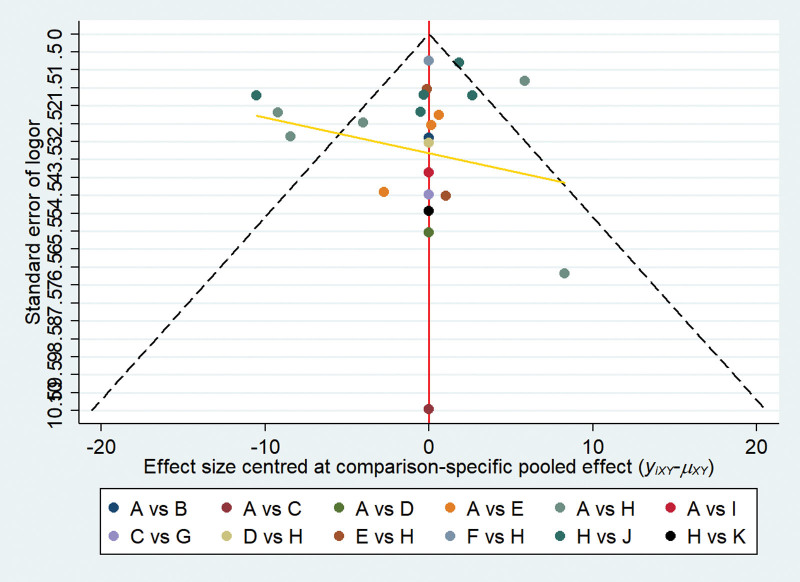
Funnel plot of THI. THI = tinnitus handicap inventory.

All 36 studies mentioned randomized grouping, 24 studies described specific methods to apply randomization, and allocation concealment was reported in 5 studies. Six articles mentioned that used blinding were not blinding of participants and personnel, but rather blinding of outcome assessment. Five articles described follow-up bias and provided a detailed explanation of the exclusion, data from other studies are complete. None of the included studies mentioned reporting bias or other biases. Specific results are shown in Figures 2 and 3. Funnel plot of the publication bias (Figs. 4 and 5) revealed general symmetry.

### 3.3. Primary outcome: Response rate

**Figure 6.**
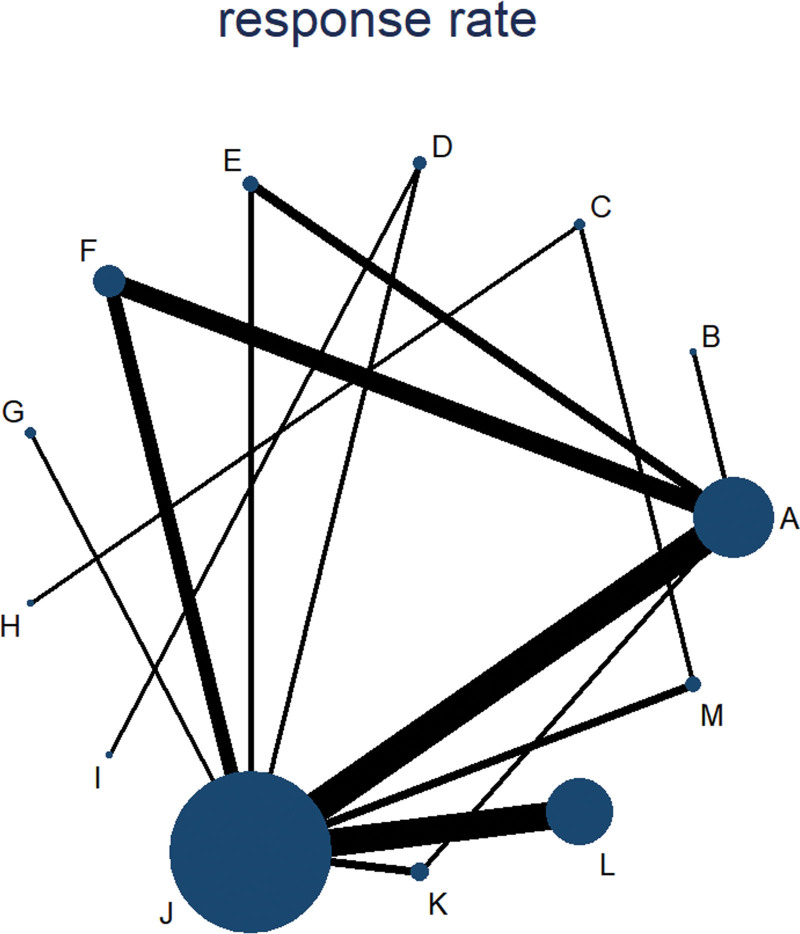
Network diagram (response rate). Interventions: (A) acupuncture, (B) moxibustion, (C) electroacupuncture, (D) acupoint injection, (E) warm acupuncture, (F) acupuncture and moxibustion, (G) acupuncture and acupoint injection, (H) electroacupuncture and warm acupuncture, (I) acupoint injection and warm acupuncture, (J) western medical treatment, (K) acupuncture and western medical treatment, (L) acupoint injection and western medical treatment, and (M) electroacupuncture and western medical treatment.

**Figure 7.**
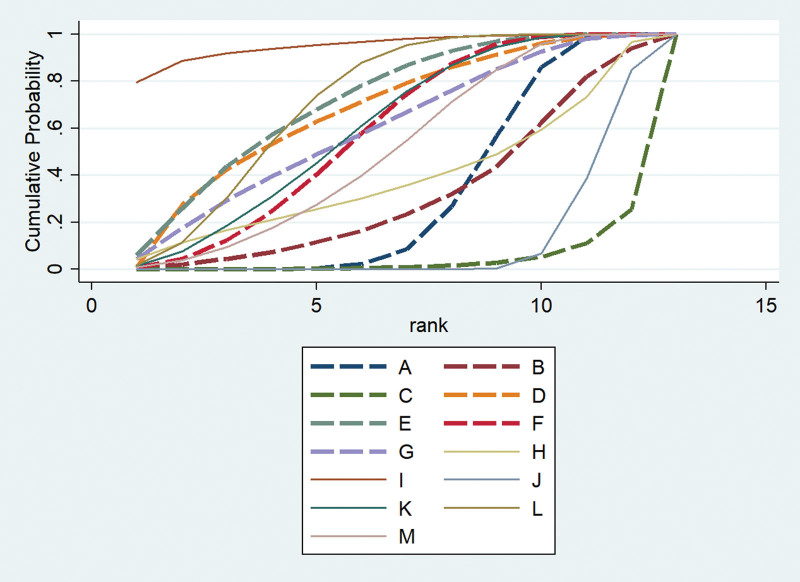
SUCRA for response rate.

Thirty-four studies involving 2489 participants evaluated response rate. Network plots of the eligible comparisons for response rate are shown in Figure 6. We used A for acupuncture, B for moxibustion, C for electroacupuncture, D for acupoint injection, E for warm acupuncture, F for acupuncture and moxibustion, G for acupuncture and acupoint injection, H for electroacupuncture and warm acupuncture, I for acupoint injection and warm acupuncture, J for western medical treatment, K for acupuncture and western medical treatment, L for acupoint injection and western medical treatment, M for electroacupuncture and western medical treatment. The results of the NMA showed significant differences in the 9 acupuncture-related treatments, when compared to the use of western medical treatment alone (OR 2.03, 95%CI (1.34, 3.07) for A; OR 4.47, 95%CI (1.30, 15.41) for D; OR 4.61, 95%CI (1.79, 11.87) for E; OR 3.43, 95%CI (1.87, 6.39) for F; OR 3.63, 95%CI (1.08, 12.18) for G; OR 16.76, 95%CI (2.56, 109.62) for I; OR 3.58, 95%CI (1.73, 7.42) for K; OR 4.38, 95%CI (2.92, 6.57) for L; OR 2.89, 95%CI (1.23, 6.83) for M). Compared with A, there are significant differences in 2 acupuncture-related treatments (OR 0.12, 95%CI (0.02, 0.83) for I; OR 0.46, 95%CI (0.26, 0.83) for L). Compared with C, there are significant differences in 7 acupuncture-related treatments (OR 0.11, 95%CI (0.02, 0.83) for D; OR 0.11, 95%CI (0.02, 0.68) for E; OR 0.15, 95%CI (0.03, 0.79) for F; OR 0.03, 95%CI (0.00, 0.35) for I; OR 0.14, 95%CI (0.02, 0.79) for K; OR 0.11, 95%CI (0.02, 0.58) for L; OR 0.17, 95%CI (0.05, 0.65) for M) (Suppl. Table 3, http://links.lww.com/MD/J676). The consistency test showed that the consistency was good (*P >* .05) (Suppl. Table 4a, http://links.lww.com/MD/J677). The SUCRA indicated acupoint injection combined with warm acupuncture was the most effective for response rate, followed by warm acupuncture and acupoint injection combined with western medical treatment (Fig. 7).

### 3.4. Secondary outcome: THI

**Figure 8.**
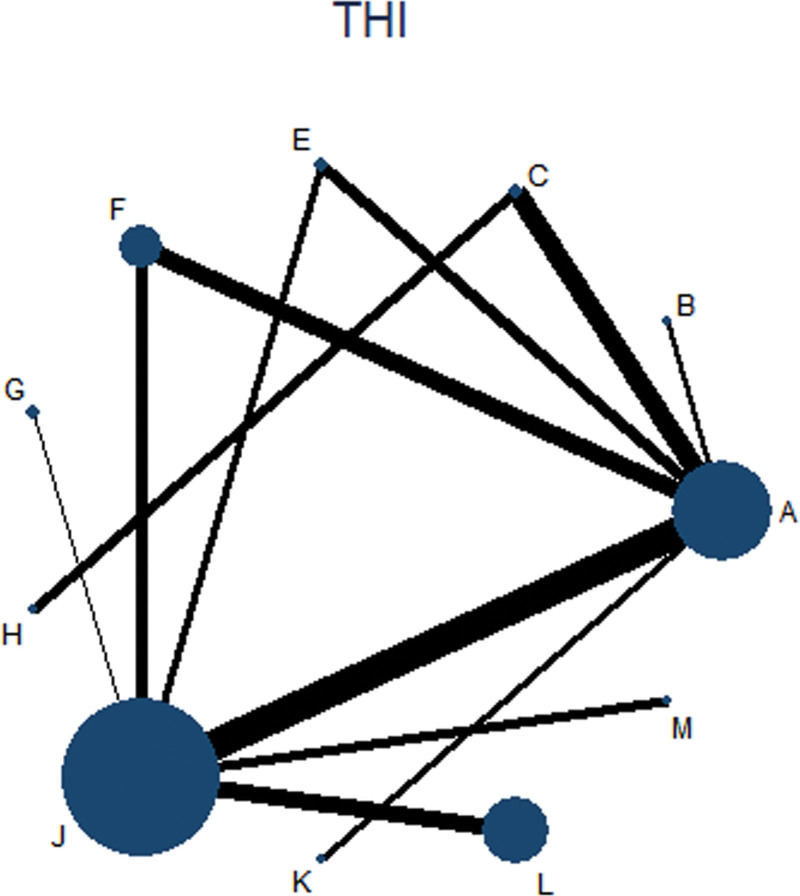
Network diagram (THI). THI = tinnitus handicap inventory.

**Figure 9.**
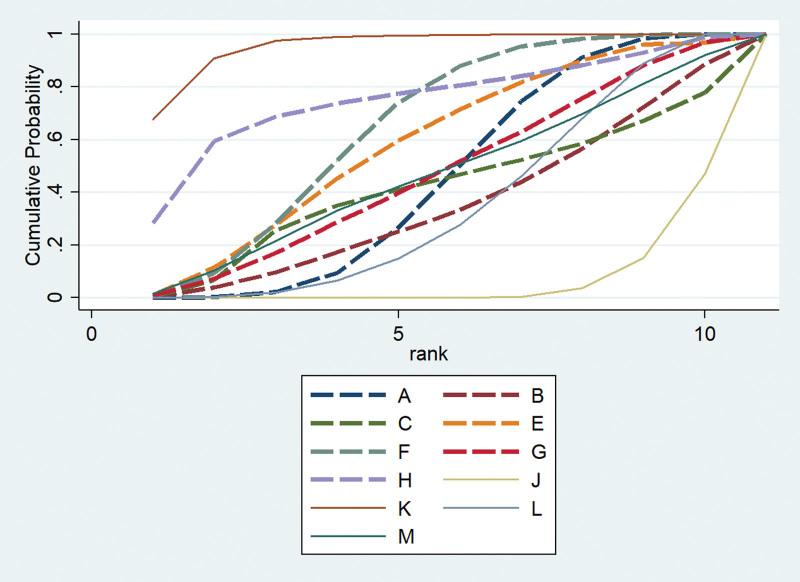
SUCRA for THI. THI = tinnitus handicap inventory.

Twenty-three studies involving 1588 participants evaluated THI. Network plots of the eligible comparisons for THI are shown in Figure 8. The results of the NMA showed significant differences in the 3 acupuncture-related treatments, when compared to the use of western medical treatment alone (OR 9458.23, 95%CI (79.69, 1.12e + 06) for A; OR 132627.73, 95%CI (7.66, 2.30e + 09) for E; OR 307942.56, 95%CI (6616.37, 1.54e + 08) for F; OR 2.52e + 12, 95%CI (2.12e + 06, 2.99e + 18) for K; OR 1373.01, 95%CI (9.02, 209026.33) for L). Compared with L, there is significant difference in 1 acupuncture-related treatment (OR 1.83e + 09, 95%CI (645.36, 5.21e + 15) for K). Compared with K, there are significant differences in 5 acupuncture-related treatment (OR 0.00, 95%CI (0.00, 0.00) for A; OR 0.00, 95%CI (0.00, 0.02) for B; OR 0.00, 95%CI (0.00, 0.79) for E; OR 0.00, 95%CI (0.00, 0.22) for F; OR 0.00, 95%CI (0.00, 0.21) for G). (Suppl. Table 3, http://links.lww.com/MD/J676). The consistency test showed that the consistency was good (*P >* .05) (Suppl. Table 4b, http://links.lww.com/MD/J678). The SUCRA indicated acupuncture combined with western medical treatment was the most effective for THI, followed by electroacupuncture combined with warm acupuncture and acupuncture combined with moxibustion (Fig. 9).

## 4. Discussion

Tinnitus is a heterogeneous condition associated with anxiety, depression, stress, cognitive impairment and sleep disorders.^[20]^ The pathogenesis of tinnitus is complicated, some researchers believe that the abnormal electrical activity of nerve fibers in the auditory conduction pathway is the basis of tinnitus, and the limbic system and autonomic nervous system are involved in the formation of tinnitus.^[21,22]^ Although many treatment approaches such as counseling, cognitive behavioral therapy, tinnitus retraining therapy, hearing aids and pharmacological therapy are available, the efficacy of most interventions for tinnitus remains unclear. As an important component of Chinese medicine, acupuncture has unique advantages in the treatment of tinnitus. In this study, we evaluated the relative efficacy of different acupuncture therapies in tinnitus patients in a more comprehensive manner which differs from the traditional meta-analysis.

The main findings of the current NMA were that acupoint injection combined with warm acupuncture was the most effective for response rate, followed by warm acupuncture and acupoint injection combined with western medical treatment. Acupuncture combined with western medical treatment was the most effective for THI, followed by electroacupuncture combined with warm acupuncture and acupuncture combined with moxibustion.

Acupoint injection combined with warm acupuncture as the best option for improving the clinical effectiveness of tinnitus, similar results were obtained in Hui Li study,^[23]^ which concluded that acupoint injection was more effective than intravenous and intratympanic injections in the treatment of tinnitus. Acupoint injection is an alternative supplementary therapy, it is based on the Chinese meridian theory, the drug will be injected into specific acupoints, through the stimulation of acupoints to achieve the purpose of treatment.^[24]^ But some of the findings differ from our study,^[25]^ there may be 2 possible reasons: first, the difference of statistical methods, then the treatment group of our study included a more comprehensive acupuncture-related treatment measures, such as acupoint injection, warm acupuncture and so on, which is an innovation in our study and provides evidence-based medical evidence for the promotion and application of acupuncture-related treatments in clinical practice.

Warm acupuncture is a combinatorial therapy of acupuncture and moxibustion, which effects of warm acupuncture consist of acupuncture effect, warming effect, and acupoint-specific stimulation and is one of the most popular traditional Chinese medicine techniques used routinely in Asian countries. The warm acupuncture has the functions of enhancing immunity, regulating blood circulation, regulating voxels, inhibiting inflammation and preventing diseases.^[26,27]^ There are relatively few studies on warm acupuncture for tinnitus, but many studies on the mechanism of warm acupuncture for insomnia. since tinnitus is closely associated with insomnia. Many studies of people with tinnitus demonstrate the frequent occurrence of sleep problems in general.^[28]^ Studies have suggested that the warm acupuncture intervention could regulate a variety of microbial genera and metabolites related to insomnia.^[29]^ Other studies have shown that warm acupuncture can improve brain neurotransmitters in people with insomnia.^[30]^ Therefore, warm acupuncture may work indirectly by improving sleep, but the mechanism needs to be further studied. Some studies have shown that warm needle therapy is more suitable for deficiency evidence.^[31]^There are many TCM syndromes of tinnitus, such as liver fire disturbing upward, deficiency of spleen and stomach and deficiency of kidney essence. However, this study did not standardize TCM syndromes of tinnitus patients included in the literature, which may affect the results. Therefore, it is very important to treat based on syndrome differentiation, and not to stick to 1 method.

If the THI score of patients is high, acupuncture combined with western medical treatment, electroacupuncture combined with warm acupuncture or acupuncture combined with moxibustion could be chosen. THI was able to predict emotional changes related to depression, anxiety, and stress. These results confirm the importance of the THI in evaluating patient with tinnitus.^[32]^ Studies have shown that tinnitus correlates with symptoms of depression, anxiety, and somatization.^[33,34]^ Clinicians should be aware of this and assist patients in receiving comprehensive care.

In this study we found that western medical treatments was more beneficial in improving tinnitus when combined with acupuncture or acupoint injection treatment. Although pharmacological is not recommended in the guideline,^[14]^ the vast majority of western medical treatments in this study were pharmacological, in addition to including sound therapy, hyperbaric oxygen, educational counseling, and cognitive behavioral therapy, which may have some impact on the results, because there are studies showing that different western medical treatments in the treatment of tinnitus is different,^[35,36]^ but this is, after all, an optimistic result. Acupuncture combined with western medicine may provide better results in the treatment of tinnitus, pharmacological with brain-acting effect (for example, amitriptyline, acamprosate, and gabapentin) or anti-inflammation/anti-oxidant effect (for example, intra-tympanic dexamethasone injection plus oral melatonin) can be a better option.^[37]^

Our present study differs from previous studies in that we have used a NMA to compare the differences in efficacy between different acupuncture methods and with conventional western medicine therapy through direct and indirect comparisons, we can not only draw the conclusion that acupuncture is better than conventional western medicine in the treatment of tinnitus, but also draw the conclusion that which kind of acupuncture is better. And we searched more databases at home and abroad, which can provide more specific and detailed clinical guidance on the choice of acupuncture therapy and the selection of different acupuncture modalities depending on the patient condition.^[12,38]^

Several potential limitations should be considered. Above all, this NMA may have been underpowered due to the heterogeneity of the participants (e.g., comorbidities, baseline severity of tinnitus, duration of tinnitus onset, treatment duration in each study and follow-up duration). And tinnitus is a subjective symptom and there is no objective measurement to measure its severity or determine the effectiveness of its treatment,^[39]^ tinnitus efficacy was assessed using a subjective questionnaire, which may lead to heterogeneity of the reported results, this was an unavoidable limitation of any NMA investigating subjective outcomes. Objective measures of tinnitus are also being researched,^[40,41]^ and the results of these studies may lead to a major breakthrough in the diagnosis and management of tinnitus. In the second place, the specificity and complexity of the acupuncture-related treatment limits the application of the blinding of participants and intervenors, which may have some impact on the efficacy, as tinnitus is a condition closely related to psychological factors,^[42]^ and psychological comfort cannot be excluded. Thirdly, given the relatively small number of patients and RCTs, the main findings of this study should probably be conservatively applied to clinical practice. In the end, we did not investigate adverse events, because there are too few studies providing data on adverse events to form a network. In addition, to further clarify the better treatments to the tinnitus patients, more rigorous RCT researches with high grade evidence and greater sample sizes that include direct comparisons for different acupuncture-related treatments are needed, we still need to update the literature to incorporate higher quality studies in the future to find more effective interventions for tinnitus.

## 5. Conclusion

This NMA found acupuncture combined with western medical treatment, warm acupuncture, acupoint injection combined with warm acupuncture, acupoint injection combined with western medical treatment, electroacupuncture combined with warm acupuncture and acupuncture combined with moxibustion were associated with superior improvement in tinnitus severity and response rate compared to western medical treatment alone in tinnitus patients without specific or treatable origin. Acupuncture seems to be a better trend treatment for tinnitus. However, because some of the intervention comparisons were based on only a few RCTs and the severity of tinnitus is still a subjective measurement, clinicians should be more cautious about the results of our study. Further rigorous RCT studies that include direct comparisons for different acupuncture-related treatments, are encouraged to provide the most promising evidence for patients with tinnitus.

## Author contributions

**Data curation:** Lin Ji, Haopeng Zhang.

**Formal analysis:** Lin Ji, Haopeng Zhang, Lihua Wang.

**Investigation:** Haopeng Zhang, Lihua Wang, Ziming Yin, Jingtu Cen.

**Resources:** Lin Ji, Haopeng Zhang, Lihua Wang.

**Visualization:** Lin Ji, Lihua Wang.

**Writing – original draft:** Lin Ji.

**Writing – review & editing:** Yu Guo.

## Supplementary Material










