# Supplementary material for: Network meta-analysis of acupuncture for tinnitus
Source: Medicine (Baltimore). 2023 Sep 29;102(39):e35019. doi: 10.1097/MD.0000000000035019 (PMC10545278; doi:10.1097/MD.0000000000035019)
Supplement: Supplementary file 2 [file medi-102-e35019-s002.docx]

**Suppl. Table 2: characteristics of the included studies.**

|  | Study name | Limitations on the duration of tinnitus at the time of inclusion | Comparison  (treatment and control) | Samples（n） | Age(year)  （Mean±SD） | Female  (n) | Treatment duration | THI | response (n) |
| --- | --- | --- | --- | --- | --- | --- | --- | --- | --- |
| 1 | Qin Tingting(2022)^[1]^ | 1M-3Y | H | 29 | 38.48±11.49 | 16 | 4W | 7.14±3.94 | 26 |
|  |  |  | C | 30 | 40.57±12.60 | 14 |  | 10.03±4.12 | 21 |
| 2 | Chen Jiaofeng(2022)^[2]^ | NR | K | 34 | 47±17 | 16 | 4W | NR | 27 |
|  |  |  | J | 34 | 48±17 | 15 |  |  | 22 |
| 3 | Hou Huifen(2022)^[3]^ | NR | L | 60 | 39.87±3.02 | 30 | 4W | 29.18±3.48 | 56 |
|  |  |  | J | 60 | 40.15±3.14 | 28 |  | 33.21±5.12 | 45 |
| 4 | Yang Jifeng(2021)^[4]^ | ＜1Y | K | 29 | 42.41±10.13 | 16 | 3W | NR | 25 |
|  |  |  | J | 28 | 42.36±9.27 | 17 |  |  | 18 |
| 5 | Chen Chan(2021)^[5]^ | NR | F | 30 | 44.03±11.63 | 12 | 1M | 23.27±17.28 | 26 |
|  |  |  | A | 30 | 42.93±12.01 | 15 |  | 32.47±16.92 | 23 |
| 6 | Yao Weijie(2021)^[6]^ | ≥6M | A | 39 | 43.96±4.01 | 25 | 4W | 29.28±5.16 | 35 |
|  |  |  | J | 39 | 43.96±4.01 | 26 |  | 49.25±6.31 | 28 |
| 7 | Guan Ningning(2021)^[7]^ | 1W-3Y | K | 30 | 44.3±13.8 | 18 | 2W | 27.34±16.28 | 27 |
|  |  |  | A | 30 | 42.6±13.3 | 16 |  | 46.74±13.46 | 20 |
| 8 | Yao Weijie(2021)^[8]^ | ≥3M | G | 50 | 35.56±3.15 | 26 | 20d | 37.88±4.17 | 46 |
|  |  |  | J | 50 | 34.86±3.22 | 27 |  | 47.16±3.33 | 38 |
| 9 | Zou Miyi(2021)^[9]^ | ≥1M | F | 25 | 44.04±6.88 | 12 | 20d | NR | 23 |
|  |  |  | J | 25 | 46.44±8.87 | 15 |  |  | 19 |
| 10 | Li Yaxue(2021)^[10]^ | ≤1Y | A | 54 | 46.78±7.93 | 31 | 2W | NR | 46 |
|  |  |  | J | 27 | 44.93±8.65 | 13 |  |  | 20 |
| 11 | Lin Youqing(2020)^[11]^ | NR | I | 33 | 43.61±8.94 | 18 | 32d | 36.42±9.93 | 30 |
|  |  |  | D | 33 | 42.52±8.486 | 16 |  | 46.15±6.59 | 24 |
| 12 | Hu Wei(2020)^[12]^ | ＜1Y | F | 30 | 41.77±12.387 | 14 | 4W | 34.20±17.75 | 22 |
|  |  |  | J | 29 | 42.38±11.324 | 18 |  | 42.41±16.87 | 18 |
| 13 | Tian Li(2019)^[13]^ | ≥1M | L | 40 | 40.9±2.3 | 25 | 2W | 29.5±10.6 | 37 |
|  |  |  | J | 40 | 41.4±2.7 | 24 |  | 35.9±8.7 | 23 |
| 14 | Chen Xiao(2019)^[14]^ | ≤3M | L | 32 | 40.25±11.27 | 15 | 2W | 31.5±7.2 | 26 |
|  |  |  | J | 32 | 42.78±12.84 | 20 |  | 34.74±6.54 | 22 |
| 15 | Yang Yang(2019)^[15]^ | ≤3M | L | 81 | 41.06±8.09 | 38 | 2W | NR | 67 |
|  |  |  | J | 81 | 43.23±8.86 | 35 |  |  | 47 |
| 16 | Song Chunxia(2019)^[16]^ | NR | F | 33 | 41.9±9.4 | 16 | 3W | 31.46±5.68 | 30 |
|  |  |  | J | 33 | 42.5±8.6 | 15 |  | 38.53±6.8 | 23 |
| 17 | Zhong Yumei(2018)^[17]^ | ≥1M | E | 30 | 44±9 | 16 | 4W | 16.8±20.06 | 26 |
|  |  |  | A | 30 | 43±10 | 17 |  | 23.47±22.68 | 22 |
| 18 | Liang Xiulang(2018)^[18]^ | NR | A | 30 | 39.56±12.54 | 13 | 30d | 16.63±8.68 | 26 |
|  |  |  | J | 30 | 42.24±10.26 | 14 |  | 26.75±10.34 | 19 |
| 19 | Mohammad Berjis Ghahfarokhi(2018)^[19]^ | ≥2M | A | 30 | 50.61±11.29 | 11 | 4W | 43.61±24.414 | NR |
|  |  |  | J | 17 | 52.35±11.25 | 7 |  | 66±20.530 |  |
| 20 | Bong Hyun Kim(2017)^[20]^ | ＞2W | C | 27 | 54.26±13.51 | 5 | 4W | 42.66±24.24 | NR |
|  |  |  | A | 12 | 49.33±5.57 | 6 |  | 41.83±32.46 |  |
| 21 | Shen Mingxue(2017)^[21]^ | NR | F | 36 | 47.14±9.04 | 17 | 6W | 26.50±9.51 | 34 |
|  |  |  | A | 36 | 48.67±7.21 | 21 |  | 32.33±9.72 | 31 |
| 22 | Yan Rubing(2017)^[22]^ | NR | L | 58 | 45.8±10.2 | 29 | 36d | NR | 53 |
|  |  |  | J | 58 | 47.3±9.5 | 30 |  |  | 41 |
| 23 | Qu Zhongyuan(2017)^[23]^ | ≤3M | L | 70 | 35.34±10.36 | 37 | 2W | 25.23±9.29 | 65 |
|  |  |  | J | 70 | 34.13±11.72 | 34 |  | 41.62±11.02 | 54 |
| 24 | Zhou Xin(2017)^[24]^ | ≤3M | L | 35 | 49.97±8.78 | 15 | 2W | 29.57±6.59 | 33 |
|  |  |  | J | 35 | 47.4±7.31 | 17 |  | 35.77±7.56 | 14 |
| 25 | Zhu Mengdie(2017)^[25]^ | NR | M | 30 | 47.4±14.2 | 17 | 7d | 33.33±18.96 | 25 |
|  |  |  | J | 30 | 46.3±14.5 | 12 |  | 42.47±19.21 | 20 |
| 26 | Duan Shengde(2016)^[26]^ | ≥3M | E | 26 | 43±15 | 12 | 4W | 19.03±10.68 | 24 |
|  |  |  | J | 27 | 42±15 | 11 |  | 28.22±11.371 | 22 |
| 27 | Zhuang Keqing(2016)^[27]^ | NR | B | 31 | 54.7±9.4 | 21 | 3W | 29.96±13.16 | 23 |
|  |  |  | A | 30 | 50.2±11.1 | 18 |  | 27.54±9.11 | 23 |
| 28 | Zhang Zhigang(2016)^[28]^ | NR | A | 30 | 41.2±6.4 | 13 | 30d | 25.4±9.3 | 21 |
|  |  |  | J | 30 | 42.5±6.9 | 15 |  | 30.3±7.6 | 16 |
| 29 | Dai Wei (2015)^[29]^ | NR | E | 30 | 52.3±2.5 | 14 | 2M | NR | 28 |
|  |  |  | A | 30 | 54.5±4.0 | 12 |  |  | 24 |
| 30 | Zheng Xiaoyan(2015)^[30]^ | ≤1Y | A | 20 | 47.35±3.96 | 11 | 30d | NR | 17 |
|  |  |  | J | 20 | 47.35±4.36 | 12 |  |  | 15 |
| 31 | Gao Yunzhu(2015)^[31]^ | 0.5Y-3Y | A | 30 | 39.4±6.33 | 14 | 4W | 18.67±10.8 | 26 |
|  |  |  | J | 30 | 40.1±6.08 | 13 |  | 24.34±11.34 | 22 |
| 32 | Jiang Bin(2015)^[32]^ | NR | A | 50 | 69.88±5.9 | 39 | 6W | NR | 46 |
|  |  |  | J | 50 | 70.82±7.3 | 40 |  |  | 39 |
| 33 | Wang Xi(2014)^[33]^ | NR | M | 27 | 32±15 | 1 | 10d | NR | 23 |
|  |  |  | C | 26 | 31±17 | 15 |  |  | 13 |
| 34 | Zhang Yun(2013)^[34]^ | ≤2M | F | 30 | 43.63±12.22 | 19 | 5W | 18.01±8.42 | 29 |
|  |  |  | A | 30 | 41.07±14.87 | 16 |  | 24.33±11.11 | 26 |
| 35 | Wang Lin(2013)^[35]^ | NR | M | 30 | 50±4 | 14 | 20d | NR | 25 |
|  |  |  | J | 30 | 51±5 | 13 |  |  | 18 |
| 36 | Wu Hongjie(2012)^[36]^ | NR | D | 37 | 43.58±15.67 | 21 | 10d | NR | 33 |
|  |  |  | J | 37 | 44.07±16.31 | 19 |  |  | 24 |

Abbreviation: NR: not reported; THI: Tinnitus Handicap Inventory; A: acupuncture; B: moxibustion; C: electroacupuncture; D: acupoint injection; E: warm acupuncture; F: acupuncture and moxibustion; G: acupuncture and acupoint injection; H: electroacupuncture and warm acupuncture; I: acupoint injection and warm acupuncture; J: western medical treatment; K: acupuncture and western medical treatment; L: acupoint injection and western medical treatment; M: electroacupuncture and western medical treatment; W: week; M: month; Y: Year.

**References**

1. Qin Tingting, *Clinical study on treatment of tinnitus of qi stagnancy and blood stasis syndrome with electro-acupuncture combined with ginger partition moxibustion*. 2022, Nanjing University of Chinese Medicine.

2. Chen Jiaofeng, *Scraping needling technique combined with western medication for neurogenic tinnitus of kidney essence deficiency:a randomized controlled trial.* Chinese Acupuncture & Moxibustion, 2022. **42**(9): p. 991-994.

3. Hou Huifen, *Clinical effect of acupoint injection of lidocaine combined with gastrodin in the treatment of idiopathic tinnitus.* Chinese Medical Digest: Otorhinolaryngology, 2022. **36**(1): p. 83-85.

4. Yang, J., *Clinical Observation of Acupuncture with Yuan-Source and Luo-Connecting Acupoints Combined with Ginaton and Mecobalamine in The Treatment of Liver Fire Disturbance Type Tinnitus*. 2021, Fujian University of Chinese Medicine.

5. Chen Chan, *Observation on the clinical effect of ironing moxibustion combined with acupuncture in treating primary tinnitus*. 2021, Anhui University of Chinese Medicine.

6. Yao Weijie, *Clinical observation on treatment of Idiopathic tinnitus with liverfire up-stirring type.* JOURNAL OF PRACTICAL TRADITIONAL CHINESE MEDICINE, 2021. **37**(9): p. 1572-1574.

7. Guan Ningning, *Clinical effect analysis of a comprehensive treatment in the tinnitus patients with the syndrome of liver qi stagnation.* Journal of Changchun University of Chinese Medicine, 2021. **37**(5): p. 1030-1033.

8. Yao Weijie, *Clinical effect of acupuncture combined with acupoint injection on phlegm-fire stagnation tinnitus.* Chinese Journal of Practical Medicine, 2021. **48**(14): p. 111-114.

9. Zou Miyi, *Clinical observation of reed tube moxibustion combined with acupuncture in treating primary tinnitus.* CHINA’S NATUROPATHY, 2021. **29**(7): p. 49-51.

10. Li Yaxue, *Clinical Observations on the Efficacy of Midnight-Midday Ebb Flow Acupuncture Combined with Points Selection Based on Syndrome Differentiation in Treating Tinnitus of Flaring up of Liver-Fire.* Information on Traditional Chinese Medicine, 2021. **38**(2): p. 49-52.

11. Lin Youqing, *Clinical observation on tinnitus of Kidney Essence Deficiency Stasis syndrome by warm moxibustion combined with acupoint injection*. 2020, Guangzhou University of Chinese Medicine.

12. Hu Wei, *Clinical Observation of Acupuncture Combined with Baihui Moxibustion in the Treatment of Primary Tinnitus of Spleen and Stomach Weakness Type*. 2020, Fujian University of Chinese Medicine.

13. Tian Li, *Application of Acupoint Injection of Auricular Vagal Root in the Treatment of Idiopathic Tinnitus.* Journal of Ningxia Medical University, 2019. **41**(9): p. 947-950.

14. Chen Xiao, *Clinical Study on Salvia Injection at Tinggong Acupoint for Acute Subjective Tinnitus.* JOURNAL OF NEW CHINESE MEDICINE, 2019. **51**(12): p. 201-203.

15. Yang Yang, *81 cases of primary tinnitus treated by acupuncture point injection at the posterior hydrophobia point of asparagine.* HUNAN JOURNAL OF TRADITIONAL CHINESE MEDICINE, 2019. **35**(5): p. 84-85.

16. Song Chunxia, *Acupuncture with thunder fire moxibustion for the treatment of phlegm and internal stagnation Clinical study of neurogenic tinnitus.* JOURNAL OF HEBEI TCM AND PHARMACOLOGY, 2019. **34**(1): p. 43-46.

17. Zhong Yumei, *Clinical Observations on Reed Tube Moxibustion plus Acupuncture for Nervous Tinnitus.* Shanghai J Acu-mox, 2018.

18. Liang Xiulang, *Clinical Study on Treatment of Nervous Tinnitus by Acupuncture method of TiaoGanShengYangYiQi*. 2018, Guangxi University of Chinese Medicine.

19. Ghahfarokhi, M.B., *Clinical Observation on the Efficacy of Acupuncture Treatment for Tinnitus*. 2018, Beijing University of Chinese Medicine.

20. Kim, B.H., K. Kim, and H.J. Nam, *A comparative study on the effects of systemic manual acupuncture, periauricular electroacupuncture, and digital electroacupuncture to treat tinnitus: a randomized, paralleled, open-labeled exploratory trial.* 2017. **17**(1): p. 85.

21. Shen Mingxue, *Tianzhu moxibustion combined with the clinical*

*research of acupuncture treatment of neurogenic*

*tinnitus*. 2017, Yunnan College of Traditional Chinese Medicine.

22. Yan Rubing, *Clinical Observation of Using Auricular Point Injection Combined with Hyperbaric Oxygen in Treating Subjective Tinnitus.* Journal of Sichuan of Traditional Chinese Medicine, 2017. **35**(5): p. 193-195.

23. Qu Zhongyuan, *Clinical Research on Chimai Luxi Penetration Needling Acupoint Injection in Treatment of Idiopathic Tinnitus.* LIAONING JOURNAL OF TRADITIONAL CHINESE MEDICINE, 2017. **44**(5): p. 1056-1058.

24. Zhou Xin, *Clinical Research of Point Injection on Acute Subjectivity Tinnitus.* JETCM, 2017. **26**(5): p. 895-897.

25. Zhu Mengdie, *A Controlled Clinical Study:Analyzing the ABR Results of Electric Acupuncture Combine with Western Medicine Treatment for Subjective Tinni tus.* Zhong Yi Yao Dao Bao, 2017. **23**(5): p. 87-89.

26. Duan Shengde, *Clinical observations on acupuncture treatment of tinnitus under the theory of kidney and sanjiao communication.* Shanghai J Acu-mox, 2016. **35**(4): p. 440‐442.

27. Zhuang Keqing, *Ear tubes moxibustion clinical observation for the treatment of neurogenic tinnitus*. 2016, Nanjing University of Chinese Medicine.

28. Zhang Zhigang, *Therapeutic effect of acupuncture Jiaji Point combined with Chinese medicine on neuronal tinnitus.* CJGMCM, 2016. **31**(4): p. 550-551.

29. Dai Wei, *Efficacy of warm acupuncture in the treatment of neurogenic tinnitus caused by deficiency of qi and blood.* Chinese Manipulation & Rehabilitation Medicine, 2015: p. 233-234.

30. Zheng Xiaoyan, *Observation of the clinical efficacy of acupuncture treatment in neurogenic tinnitus of qi stagnation and blood stasis type*. 2015, Fujian University of Chinese Medicine.

31. Gao Yunzhu, *Needle head acupuncture point yunting area week with ear acupuncture point treatment clinical observation of nervous tinnitus*. 2015, Heilongjiang University of Chinese Medicine.

32. Jiang Bin, *Clinical study on syndrome differentiation combined with acupuncture in treating elderly sensorineural tinnitus.* SH. J. TCM Aug., 2015. **49**(8): p. 47-49.

33. Wang Xi, *Electroacupuncture with hyperbaric oxygen for neurogenic tinnitus 27 cases.* JOURNAL OF PRACTICAL TRADITIONAL CHINESE MEDICINE, 2014. **30**(3): p. 219.

34. Zhang Yun, *The Clinical Research for Tinnitus of Deficiency Qi and Blood by Moxibustion of Moxa Cone on Dazhui (DU 14) Therapy*. 2013, Guangzhou University of Chinese Medicine.

35. Wang Lin, *Observations on the Efficacy of Acupuncture plus Medicine in Treating Tinnitus.* Shanghai J Acu-mox, 2013. **32**(2): p. 112-114.

36. Wu Hongjie, *Analysis of lidocaine acupoint injection therapy for simple neurogenic tinnitus.* CHINA FOREIGN MEDICAL TREATMENT, 2012. **31**(9): p. 109,111.
