# Supplementary material for: Network meta-analysis of acupuncture for tinnitus
Source: Medicine (Baltimore). 2023 Sep 29;102(39):e35019. doi: 10.1097/MD.0000000000035019 (PMC10545278; doi:10.1097/MD.0000000000035019)
Supplement: Supplementary file 3 [file medi-102-e35019-s003.docx]

**Suppl. Table 3: League table of the response rate and improvement of severity of tinnitus.**

| A | 11.25 (0.00,2.14e+06) | 2.27 (0.00,2.22e+10) | - | 0.07 (0.00,1574.35) | 0.03 (0.00,10.48) | 0.88 (0.00,124135.01) | 0.00 (0.00,6.67e+06) | - | **0.00 (0.00,0.00)** | 6.89 (0.01,7030.77) | 1.00 (0.00,4.07e+06) | **9458.23 (79.69,1.12e+06)** |
| --- | --- | --- | --- | --- | --- | --- | --- | --- | --- | --- | --- | --- |
| 1.14 (0.36,3.67) | B | 0.20 (0.00,4.02e+10) | - | 0.01 (0.00,43546.66) | 0.00 (0.00,1958.82) | 0.08 (0.00,1.86e+06) | 0.00 (0.00,8.19e+06) | - | **0.00 (0.00,0.02)** | 0.61 (0.00,730703.00) | 0.09 (0.00,2.56e+07) | 841.04 (0.00,3.95e+08) |
| 4.03 (0.80,20.39) | 3.53 (0.48,26.01) | C | - | 0.03 (0.00,2.47e+09) | 0.01 (0.00,2.75e+08) | 0.39 (0.00,6.75e+10) | 0.00 (0.00,6.93) | - | 0.00 (0.00,532.35) | 3.04 (0.00,8.25e+10) | 0.44 (0.00,4.21e+11) | 4171.54 (0.00,6.67e+13) |
| 0.45 (0.12,1.67) | 0.40 (0.07,2.29) | **0.11 (0.02,0.83)** | D | - | - | - | - | - | - | - | - | - |
| 0.44 (0.18,1.08) | 0.39 (0.09,1.68) | **0.11 (0.02,0.68)** | 0.97 (0.20,4.60) | E | 0.43 (0.00,28121.30) | 12.37 (0.00,2.70e+07) | 0.00 (0.00,5.68e+08) | - | **0.00 (0.00,0.79)** | 96.60 (0.00,5.63e+06) | 14.42 (0.00,5.52e+08) | **132627.73 (7.66,2.30e+09)** |
| 0.59 (0.31,1.14) | 0.52 (0.14,1.97) | **0.15 (0.03,0.79)** | 1.30 (0.33,5.22) | 1.35 (0.45,3.99) | F | 28.73 (0.00,7.74e+06) | 0.00 (0.00,4.07e+08) | - | **0.00 (0.00,0.22)** | 224.28 (0.08,660639.00) | 32.59 (0.00,2.21e+08) | **307942.56 (616.37,1.54e+08)** |
| 0.56 (0.16,2.01) | 0.49 (0.09,2.76) | 0.14 (0.02,1.00) | 1.23 (0.22,6.95) | 1.27 (0.27,5.90) | 0.94 (0.24,3.68) | G | 0.00 (0.00,9.23e+07) | - | **0.00 (0.00,0.21)** | 7.81 (0.00,1.22e+06) | 1.13 (0.00,8.02e+07) | 10719.33 (0.21,5.54e+08) |
| 1.09 (0.13,9.41) | 0.95 (0.08,11.06) | 0.27 (0.06,1.12) | 2.39 (0.21,27.84) | 2.47 (0.24,25.13) | 1.83 (0.20,16.71) | 1.94 (0.17,22.32) | H | - | 0.00 (0.00,2.42e+09) | 466263.63 (0.00,5.08e+17) | 67746.40 (0.00,1.70e+18) | 6.40e+08 (0.00,4.41e+20) |
| **0.12 (0.02,0.83)** | 0.11 (0.01,1.00) | **0.03 (0.00,0.35)** | 0.27 (0.06,1.10) | 0.28 (0.03,2.25) | 0.20 (0.03,1.48) | 0.22 (0.02,2.02) | 0.11 (0.01,1.89) | I | - | - | - | - |
| 0.57 (0.26,1.24) | 0.50 (0.12,2.02) | **0.14 (0.02,0.79)** | 1.25 (0.30,5.24) | 1.29 (0.40,4.13) | 0.96 (0.37,2.45) | 1.01 (0.25,4.16) | 0.52 (0.06,4.91) | 4.68 (0.62,35.05) | K | **1.83e+09 (645.36,5.21e+15)** | 2.67e+08 (0.49,1.44e+17) | **2.52e+12 (2.12e+06,2.99e+18)** |
| **0.46 (0.26,0.83)** | 0.40 (0.11,1.49) | **0.11 (0.02,0.58)** | 1.02 (0.28,3.75) | 1.05 (0.38,2.94) | 0.78 (0.37,1.65) | 0.83 (0.23,2.97) | 0.43 (0.05,3.69) | 3.82 (0.56,26.12) | 0.82 (0.36,1.88) | L | 0.15 (0.00,642131.31) | **1373.01 (9.02,209026.33)** |
| 0.70 (0.27,1.82) | 0.61 (0.14,2.77) | **0.17 (0.05,0.65)** | 1.54 (0.34,6.97) | 1.59 (0.44,5.72) | 1.18 (0.41,3.42) | 1.26 (0.28,5.54) | 0.65 (0.09,4.49) | 5.79 (0.73,45.69) | 1.24 (0.40,3.82) | 1.51 (0.59,3.92) | M | 9449.74 (0.00,1.79e+10) |
| **2.03 (1.34,3.07)** | 1.77 (0.51,6.13) | 0.50 (0.10,2.41) | **4.47 (1.30,15.41)** | **4.61 (1.79,11.87)** | **3.43 (1.84,6.39)** | **3.63 (1.08,12.18)** | 1.87 (0.22,15.57) | **16.76 (2.56,109.62)** | **3.58 (1.73,7.42)** | **4.38 (2.92,6.57)** | **2.89 (1.23,6.83)** | J |
