# Supplementary material for: Network meta-analysis of acupuncture for tinnitus
Source: Medicine (Baltimore). 2023 Sep 29;102(39):e35019. doi: 10.1097/MD.0000000000035019 (PMC10545278; doi:10.1097/MD.0000000000035019)
Supplement: Supplementary file 4 [file medi-102-e35019-s004.docx]

**Suppl. Table 4a: Consistency test of the response rate.**

| Loop | IF | p_value | CI_95 | Loop_Heterog_tau^2^ |
| --- | --- | --- | --- | --- |
| A-J-K | 1.502 | .085 | (0.00,3.21) | 0.00 |
| A-E-J | .953 | .370 | (0.00,3.04) | 0.00 |
| A-F-J | .912 | .175 | (0.00,2.23) | 0.00 |
