# Supplementary material for: Network meta-analysis of acupuncture for tinnitus
Source: Medicine (Baltimore). 2023 Sep 29;102(39):e35019. doi: 10.1097/MD.0000000000035019 (PMC10545278; doi:10.1097/MD.0000000000035019)
Supplement: Supplementary file 5 [file medi-102-e35019-s005.docx]

**Suppl. Table 4b: Consistency test of THI.**

| Loop | IF | p_value | CI_95 | Loop_Heterog_tau^2^ |
| --- | --- | --- | --- | --- |
| A-E-H | 11.254 | .125 | (0.00,25.65) | 42.398 |
| A-D-H | 9.475 | .487 | (0.00,36.17) | 65.309 |
